# Opportunities and Limitations of Wrist-Worn Devices for Dyskinesia Detection in Parkinson’s Disease

**DOI:** 10.3390/s25144514

**Published:** 2025-07-21

**Authors:** Alexander Johannes Wiederhold, Qi Rui Zhu, Sören Spiegel, Adrin Dadkhah, Monika Pötter-Nerger, Claudia Langebrake, Frank Ückert, Christopher Gundler

**Affiliations:** 1 Institute for Applied Medical Informatics, University Medical Center Hamburg-Eppendorf, Martinistraße 52, 20246 Hamburg, Germany; 2 Hospital Pharmacy, University Medical Center Hamburg-Eppendorf, Martinistraße 52, 20246 Hamburg, Germany; 3 Department of Stem Cell Transplantation, University Medical Center Hamburg-Eppendorf, Martinistraße 52, 20246 Hamburg, Germany; 4 Institute of Neurology, University Medical Center Hamburg-Eppendorf, Martinistraße 52, 20246 Hamburg, Germany

**Keywords:** Parkinson’s disease, dyskinesia, wearable devices, semantic data analysis, tsfresh, machine learning, accelerometer data, supportive decision making, feature extraction, principal component analysis

## Abstract

**Highlights:**

- Sensor-driven measurements can support the assessment of dyskinesia fluctuations in clinical practice.
- Dimensional reduction of accelerometer data using PCA improves machine learning model performance.
- Semantic feature extraction enhances model generalization and predictive capability for dyskinesia detection.
- Integrating standardized neurological assessments can further improve the reliability of sensor-based monitoring.

**Abstract:**

During the in-hospital optimization of dopaminergic dosage for Parkinson’s disease, drug-induced dyskinesias emerge as a common side effect. Wrist-worn devices present a substantial opportunity for continuous movement recording and the supportive identification of these dyskinesias. To bridge the gap between dyskinesia assessment and machine learning-enabled detection, the recorded information requires meaningful data representations. This study evaluates and compares two distinct representations of sensor data: a task-dependent, semantically grounded approach and automatically extracted large-scale time-series features. Each representation was assessed on public datasets to identify the best-performing machine learning model and subsequently applied to our own collected dataset to assess generalizability. Data representations incorporating semantic knowledge demonstrated comparable or superior performance to reported works, with peak F_1_ scores of 0.68. Generalization to our own dataset from clinical practice resulted in an observed F_1_ score of 0.53 using both setups. These results highlight the potential of semantic movement data analysis for dyskinesia detection. Dimensionality reduction in accelerometer-based movement data positively impacts performance, and models trained with semantically obtained features avoid overfitting. Expanding cohorts with standardized neurological assessments labeled by medical experts is essential for further improvements.

## 1. Introduction

The optimization and fine-tuning of therapy in response to the progression of Parkinson’s disease (PD) necessitate in-hospital diagnostic assessments and evaluations by means of clinical rating scales [1,2]. The overdosing of dopaminergic drugs often evokes levodopa-induced dyskinesia (LID), an uncomfortable side effect that is characterized by uncontrolled, involuntary muscle movements of all body parts [3]. While many symptoms associated with PD may be more effectively identified through observational means, dyskinesias in the upper limbs hold significant potential for detection using wrist-worn sensors. The direct recording of health parameters provides vital insights into the ongoing progression of motor symptoms. Unlike a clinician who can only assess a snapshot of a moment, body-mounted systems are worn continuously throughout the day, offering a temporal recording of events. The acquired data is confined to the specific body part where the sensor is worn, yet it is tailored precisely to its designated tasks, thereby facilitating the capture of therapy-dependent motor fluctuations [4,5]. Subsequently, wearable devices such as smartwatches provide accessible monitoring aids, enabling the recording and tracking of movement data for understanding motor symptom variations and optimizing therapy plans [6,7,8].

Wearables offer potential benefits for observing daily fluctuations in a hospital setting, allowing an expert-guided labeling of symptoms. While various sensor-driven approaches for capturing tremor and bradykinesia have been explored, there is a limited focus on dyskinesia detection despite its potential as a key biomarker for therapy responses [9,10]. Alongside other reports, studies conducted by Hssayeni et al. and Pfister et al. aim to assess dyskinesia in a free-living environment to be able to monitor the disease condition at home [11,12]. Hssayeni et al. seeks to estimate dyskinesia using accelerometer data during daily activities, reporting a Pearson correlation within the range of 0.70 to 0.84. In a similar vein, Pfister et al. reports the capability to detect dyskinesias in a free-living environment, achieving a sensitivity/specificity of 0.64/0.89. Acknowledging the importance of understanding the symptoms impact on everyday activities, there is clinical relevance in observing and estimating its occurrence during a hospital stay. When patients visit the clinic, neurologists adjust dopaminergic pharmaceuticals, leading to the frequent appearance of LID [3,13]. Monitoring these side effects during hospital admission could provide insights into the symptomatic fluctuations of patients, potentially aiding in the determination of the optimal drug dosage for individuals and supporting clinicians in addressing LIDs before patients are discharged. Sieberts et al. conducted a study referred to as the DREAM Challenge, which aligns with this suggestion by identifying biomarkers linked to tremor, bradykinesia, and dyskinesia in PD. Utilizing public datasets, the DREAM Challenge aimed to predict the severity of PD symptoms, resulting in an AUPR (area under the precision-recall curve) of 0.48 specifically for dyskinesia [9].

Subsequently, we encourage the implementation of a novel monitoring setup specifically designed for dyskinesias emerging during hospital admission. Additionally, we propose an evaluation of the generalizability of movement data from publicly accessible datasets to our internally collected hospital data.

Thus, this paper employs two innovative approaches for movement data representation: one is a purely semantic technique utilizing principal component analysis (PCA) in combination with biomechanical feature extraction, and the other is an automatic, serial feature representation. Following the training of these methods on publicly available datasets from the Michael J. Fox Foundation (MJFF) [14], our objective was to assess the performance of the resulting models on our own collected movement data and thus the models’ generalizability. To fulfill our objective, we formulated two intents: (1) investigating the impact of various movement data representations on model performance and (2) evaluating the generalizability of machine learning (ML) models from publicly available datasets [14] onto the PACMAN (Parkinson’s Clinical Movement Assessment) dataset.

## 2. Materials and Methods

### 2.1. Data

#### 2.1.1. Public Datasets

We used two distinct and publicly available datasets from the MJFF: the Levodopa Response Study (LRS) and the Clinician Input Study (CIS-PD) [14,15]. Both studies aim to measure movement symptoms and their fluctuations of PD by means of accelerometers and according to the Unified Parkinson’s Disease Rating Scale (UPDRS). These datasets were always retrieved together and referred to as MJFF dataset.

The LRS includes 28 patients diagnosed with PD that were monitored both in-clinic, where they engaged in a battery of standard activities, and at home while performing their daily activities. While the primary focus of the study is to comprehend motor fluctuations, patients were measured over four consecutive days with accelerometers. On the day of admission, UPDRS assessments were conducted at the clinic while patients were still on regular dopaminergic medication. Over the next two days, patients were released home, where they could carry out regular activities. On the last day, patients returned to the clinic and underwent similar UPDRS tests without any dopaminergic treatment [14].

The CIS-PD was a 6-month longitudinal investigation involving wearable tracking for 51 PD patients. The study encompassed clinic visits and at-home monitoring using smartwatches. Following the baseline assessment, in-clinic visits were scheduled at 2 weeks, 1 month, 3 months, and 6 months. During these visits, clinicians conducted standard clinical assessments and reviewed data recorded at home. Between the hospital visits, the patients were asked to continue wearing their smartwatches and regularly report symptom severity and medication intake using a mobile phone app [15].

#### 2.1.2. Data Collection of Our Own Movement Data (PACMAN)

PD patients admitted at the Department of Neurology at the University Medical Center Hamburg-Eppendorf (UKE) stay a minimum of two weeks during an inpatient care program, known as the Parkinson-Komplexbehandlung (PKB). A multidisciplinary team, including neurologists, physiotherapists, neuropsychologists, and other paramedical specialists, is dedicated to a patient-centered and individualized clinical approach in search of the optimal therapy [2,15]. This setting presents a distinctive opportunity for the continuous gathering of accelerometer data, accompanied by task-coupled severity scoring.

During our own 4-month clinical data collection, we raised 7 different standardized clinical examinations according to the third part of the Unified Parkinson Disease Rating Score (UPDRS III) per visit. An experienced neurologist decided on the examination criteria relevant for our hypothesis to detect dyskinesias during hospital admittance. The neurological task was required to not only be significant for the detection of upper limb dyskinesia but also measurable by a wrist-worn device. Hence, we decided on alternating hand movements, which include the supination and consequent pronation of the most affected hand (UPDRS 3.6). Further, we assumed that this periodic movement is projectable by semantic data representation and thus offers potential for clinically relevant feature extraction.

Each patient underwent a minimum of two daily visits over a span of up to two weeks, mirroring the duration of the PKB program [2]. During the initial consultation, the physician provided each patient with the watches and conducted the necessary setup each morning. Subsequently, the UPDRS 3.6 task was assessed, and the corresponding timestamp was annotated for accurate measurement. To ensure consistent data quality, the physician verified that the watch was worn tightly and in the correct orientation during each labeled assessment. The watch remained on the patient’s most affected side, continuously capturing accelerometer data until the physician’s return in the afternoon.

This non-interventional prospective cohort study used the accelerometer of an Apple Watch Series 6. The resulting dataset, further referred to as PACMAN, comprises movement data along with timestamped labels indicating symptom severity. The entire data collection transpired within the framework of clinical routine at an inpatient unit of the UKE and was carried out in accordance with relevant guidelines and regulations (Declaration of Helsinki). Written informed consent for the study was obtained from all participants and an approval from the Ethics Commission of the Ärztekammer Hamburg under the ID 2022-100846-BO-ff was granted beforehand.

### 2.2. Uniform Data Infrastructure

To achieve our goal of robustly detecting motor fluctuations, it was imperative to establish an infrastructure that could seamlessly integrate into clinical settings. This need was met by implementing a database adhering to the Fast Healthcare Interoperability Resources (FHIR) standard, ensuring the consistent storage and retrieval of movement data [16]. All acquired movement data is systematically deposited into the FHIR database, serving as an objective for subsequent comparative analyses. Once stored, a custom-designed data loader facilitates the retrieval of all measurements, allowing for tailored specifications and consistent loading of the requested data. Our team devised this uniform data architecture in a preceding project, with comprehensive details published elsewhere [16].

The process of obtaining all stored movement data involves a crucial consideration regarding the length of measurement samples. In the realm of sequential health data analysis, the term window size refers to the duration in which the data is examined. Given that different measurements possess unique recording lengths, the sample’s window size can be of standardized length or dynamic. Opting for a predefined length ensures straightforward cross-database compatibility but may result in an exclusion of shorter measurements. Thus, setting the window size to a smaller value may omit a necessary task characteristic in some samples [17]. Therefore, we retain the full duration of each task as observed in the clinical setup and store them as variable-length samples. All subsequently chosen methods were selected to be compatible with this kind of temporal data with varying lengths. To further ensure comparability across sessions and participants, we applied a global rotation to compensate for the different coordinate systems of the chosen wearables. Since each sample was labeled in its entirety by a clinician, it is important to preserve the full temporal context. Subsequent classification directly relies on these clinical labels and thus benefits from maintaining the integrity of the original task as far as possible.

### 2.3. Data Representations

Representing movement data is crucial for its adequate analysis. A data representation is a way of encoding information into a format interpretable by an algorithm without losing significant meaning. Therefore, the selected representation format should align with the inherent characteristics of the measured data. Employing an appropriate data representation facilitates the extraction of meaningful features during preprocessing. The resulting features are, in turn, used to train our models for the detection of dyskinesias. We opted for two distinct techniques of data representation that maintain the structure of the acquired accelerometer recordings.

#### 2.3.1. Semantic Representation: PCA and Biomechanical Features

As movement data is recorded in a three-dimensional space by accelerometers, a reduction in dimensionality is amenable to visualization and analysis through human interaction. Given our focus on the alternating hand movements, we assumed that this periodic movement should be prominent in the data. This unique characteristic provides an opportunity for the representation of multi-dimensional movement data using semantic approaches. To reduce the complexity of our data, while still respecting the activity on each axis, we utilized a PCA.

**Figure 1:**
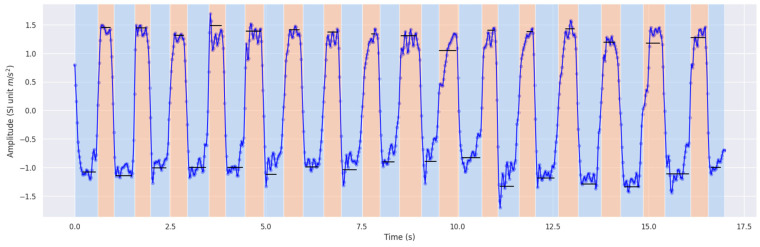
An exemplary segmented measurement. The blue segments have their extremum smaller than 0 and the orange segments show an extremum larger than 0. This segmentation is used for feature analysis.

PCA was applied to project the tri-axial accelerometer data into a single principal component, yielding a one-dimensional representation of movement over time. This projection facilitates semantic feature extraction, where distinct movement characteristics can be interpreted in a way that mirrors clinical assessment. As depicted in Figure 1, this allows clinicians and researchers alike to identify meaningful signal patterns, such as oscillation symmetry or amplitude modulation, using a reduced yet expressive representation.

Our semantic feature extraction follows the methodology introduced by Sánchez-Fernández et al., who identified 20 biomechanical features as particularly relevant for characterizing the alternating hand movement task (UPDRS 3.6) [18]. Their analysis, however, relied on multimodal sensor input (accelerometer, gyroscope, magnetometer). Since our dataset comprises only accelerometry data, we selected a subset of 9 clinically and technically interpretable features that can be derived robustly from this modality. These were selected through expert-driven, domain-specific relevance. The selected features are as follows:

- Feature for entire measurement:
  ○ Absolute mean of extremum
- Features for each part:
  ○ Number of segments comprising each part
  ○ Duration ratio of each part
  ○ Mean duration of each part
  ○ Interquartile range of each part
  ○ Relative maximum mean of each part
  ○ Relative maximum interquartile range of each part
  ○ Relative minimum mean of each part
  ○ Relative minimum interquartile range of each part

Next, to respect characteristic events of each individual measurement, we divided each sample into three distinct parts for the beginning, middle, and end. These parts are further divided into equally enduring segments. Figure 1 shows an example measurement with segments marked in orange or blue, defined by either the segment’s maxima or minima, respectively. This segmentation was crucial for capturing temporal variations within the movement, such as oscillatory or acceleration changes, which are diagnostically relevant in Parkinson’s disease.

#### 2.3.2. Automatic Feature Extraction

In our alternative representation method, we utilized an automatic time-series feature extraction tool, further referred to as tsfresh, to automate the complex process of time-series engineering. Instead of human guidance, the Python library (Version: 0.20.1 on Python 3) identifies features by considering different algorithms of signal processing and time-series analysis to extract over 3000 features from temporal structured data [19]. This vast amount of mostly interpretable features is then systematically reduced through statistical tests.

As the automatically feature extraction can be applied on both the original three-dimensional as well as the reduced data, we considered three ways of movement data representation: (1) a fully semantic technique consisting of PCA and biomechanical feature extraction, (2) the PCA combined with the automatic feature extraction, and (3) automatic feature extraction only.

### 2.4. Training of the ML Models

Each of the three representation techniques yields distinctive features for the measured UPDRS task. A vast amount of these features, especially the numerous automatically extracted features, are not relevant to our research question. However, to identify an optimal ML model that maximizes performance based on only meaningful features, we aimed to determine the best combination of each representation with available models. The core component of the resulting data pipeline is the classifier, a predefined algorithm that assigns labels based on the provided features. To identify the classifier’s highest performance, we integrated every possible combination into a grid search that predicts on the MJFF dataset.

A grid search is a systemic hyperparameter optimization that finds the optimal configuration of meaningful features, the classifier, and its hyperparameters by training each model separately [20]. Our implemented grid search used a stratified 10-fold cross validation prepared for imbalanced data [21]. We considered (1) a feature selector to find the ideal count of relevant features, (2) an oversampling technique to equalize for underrepresented labels (SMOTE) [22], and (3) different classifiers with numerate possibilities of their hyperparameters. As for the selection of parametric and non-parametric classification algorithms, we compared the performance of a logistic regression, a k-nearest neighbors’ classifier, a random forest classifier, a support vector machine, and a gradient-boosting classifier.

### 2.5. Evaluation of the Resulting ML Models

Next, we determined the ten best-performing combinations of classifiers, selectors, and samplers for each of the three methods of feature representation on the MJFF dataset. The grid search results were ranked by the unweighted F_1_ score, calculated as the arithmetic mean of all per-class F_1_ scores. By combining precision and recall into a single metric, the F_1_ score offers a balanced view of the model’s performance across both classes. Although no consensus exists on metric selection for clinical relevance in this particular task [11,12] a high F_1_ score suggests greater reliability in capturing label fluctuations, a critical requirement for potential therapeutic decision support. In addition to the F_1_ score, the accuracy of each model’s performance was also computed.

To ensure robustness of our findings, we conducted a conventional t-test, incorporating Welch’s modification to accommodate potential variations and to assess significant performance disparities among different models. We expressed our outcomes as mean ± standard deviation, with a predefined threshold for statistical significance set at *p* < 0.001.

As a final step, we employed the top 10 models per representation, based on their unweighted F_1_ performance, and implemented them on our own collected PACMAN movement data. The assessment of their performance utilized the same metric analysis and statistical significance to ensure comparability.

## 3. Results

### 3.1. The Patient Cohort

Our paper incorporated a total of 27 patients from the LRS dataset, spanning an age range of 50 to 84 years, with an average age of 67 years (±9 years). We included an average of 51 dyskinesia measurements (±9 measurements) per patient. Additionally, we included 24 patients from CIS-PD, with an average age of 63 years (±10 years), ranging from 36 to 75 years. Here, we used 12 dyskinesia measurements (±3 measurements) per patient on average. Finally, our in-clinic data collection contributed 25 patients, with an average age of 65 years (±8 years) and a range from 49 to 84 years. The PACMAN dataset incorporates an average of 3 dyskinesia measurements (±2 measurements) per patient.

### 3.2. Data Integrity

The presented data sources originate from distinct sites and were designed for different purposes. Nevertheless, they share comparability in terms of sensor type, demographics, neurological assessments, and disease-related intention of recording. The populations depicted in all MJFF studies fall within the same age range and undergo recurring hospital care for therapy adjustments. The types of sensors employed are consistent, as all studies integrate accelerometers, with both CIS-PD and PACMAN even utilizing Apple Watch devices.

Given that the MJFF studies involve a considerable number of ambulatory accelerometer recordings without precise physician annotations, our exclusive reliance on supervised labels was necessary to achieve our goal of identifying the most accurate representation of movement data for dyskinesia detection in a clinical setting. Consequently, all retained movement data and its corresponding labels are derived from hospital admittance and adhere to UPDRS standards.

### 3.3. Performance on Training Datasets

**Figure 2:**
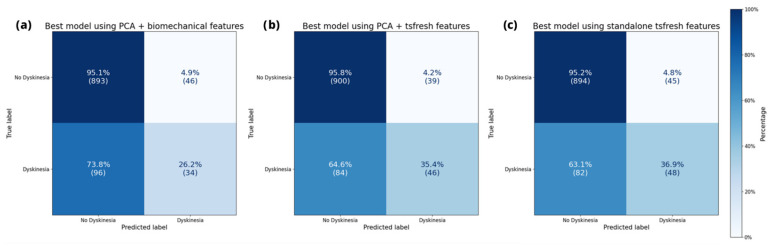
Confusion matrix of the best-performing models for each representation method on the MJFF dataset.

Figure 2 illustrates the confusion matrix of the leading model for each data representation on the MJFF studies. This visualization simultaneously presents the assigned dyskinesia label and the model’s prediction. Hence, the confusion matrix provides a reliable means to identify instances when a model incorrectly labeled a class. All depicted models adequately identified the absence of dyskinesia. However, they encountered challenges in accurately recognizing dyskinesia.

When it comes to the full evaluation of performance, we ranked the predictive outcomes for each representation by the unweighted F_1_ score. Here, we achieved peak performances of 0.68 with both data representations, employing solely automatically extracted features and the automatically extracted features on the one-dimensional representation. The semantic representation using PCA and biomechanical features yielded a slightly lower performance, registering 0.63 for unweighted F_1_. Accuracies of all three extraction methods were as high as 0.89 for each representation. The top five models per representation are listed in Table 1, Table 2 and Table 3.

Neither the purely semantic technique, nor the automatic feature extraction, alone or combined, achieved a significant difference compared to each other. Across all performed combinations, the average unweighted F_1_ yielded scores of 0.53 ± 0.05, 0.57 ± 0.08, and 0.57 ± 0.08 for PCA with biomechanical features, PCA with feature extraction, and standalone feature extraction, respectively (mean ± standard deviation).

### 3.4. Generalization into Clinical Setting

**Figure 3:**
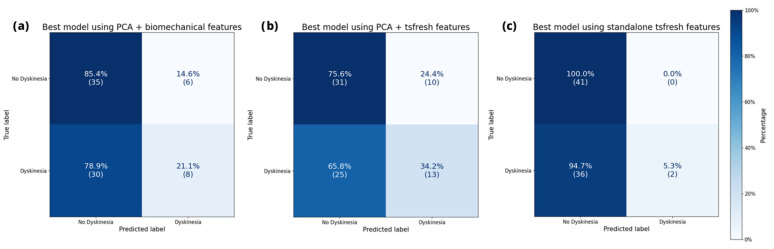
Confusion matrix of the best-performing models for each representation on the PACMAN dataset.

While the performance on the public datasets though cross validations might approximate the generalizability on unseen data, we further tested this claim by utilizing the novel clinical data collected for this study. Application of the best 10 models per representation method on the PACMAN validation set yielded nuanced findings. The confusion matrix of the best model per representation, depicted in Figure 3, shows a similar classification pattern as on the MJFF studies.

The top-performing representation for unweighted F_1_ achieved a score of 0.53, utilizing PCA in conjunction with automatically extracted features. Following this was the semantic approach with a score of 0.48, and the standalone automatically extracted features ranked the lowest with a score of 0.40. The top five outcomes per representation of our generalization efforts are detailed in Table 4, Table 5 and Table 6, including all the parameters employed.

Overall, the three methods yielded average unweighted F_1_ scores of 0.42 ± 0.04, 0.39 ± 0.06, and 0.36 ± 0.02 for PCA with biomechanical features, PCA with automatically extracted features, and automatically extracted features alone, respectively (mean ± standard deviation). Performances of the purely semantic representation were significantly higher than the automated technique (*p* < 0.001).

## 4. Discussion

This paper explores ML models for movement data representation, utilizing two distinct approaches and their combination. We first determined the top-performing models on the MJFF datasets and then evaluated their performance on our collected test dataset. Thereby, we were focusing on the impact of movement data representations and the generalizability of our resulting models for dyskinesia detection as our central hypotheses.

The results demonstrate how a dimensional reduction in movement data informed by the nature of the task has a positive impact on performance. Irrespective of whether combined with semantic or automatic extraction methods, the top 10 performing models incorporate this transformation when applied on our PACMAN dataset. The optimal performance is observed when the transformation is combined with automatic features. Nevertheless, the transformation combined with biomechanical features yielded comparable performance. This finding supports the idea that human-interpretable features enhance the ability of ML techniques to generalize across movement datasets. This finding confirms that semantically grounded preprocessing can serve as a safeguard against overfitting while supporting interpretability, a crucial factor in clinical implementation. The models using unselected features from a single automatic feature extraction generally show better results during training on the MJFF datasets but fail on our collected PACMAN data. Likely due to overfitting, it almost entirely fails to detect dyskinesia labels and assigns only two labels correctly. This underlines the importance of aligning feature representations with domain knowledge to improve robustness in real-world deployment.

Comparing the two approaches in detail, we observe that automated feature extraction (tsfresh) offers a wide array of statistical descriptors that may capture subtle signal characteristics, which contributes to strong performance on the structured and relatively homogeneous MJFF dataset. However, this approach appears to be less robust when applied to the clinically diverse PACMAN dataset, suggesting high sensitivity to variability in sensor noise, wearing conditions, and patient behavior. In contrast, the semantic representation consistently yields more stable results, even with limited and heterogeneous data. This suggests that the semantic approach not only improves interpretability for clinicians but also enhances robustness against real-world variability, which is critical for generalization across datasets. Therefore, while automated features may excel in high-data or controlled settings, semantically grounded representations prove more effective in noisy, low-data clinical environments, where reliability and explainability are essential.

Further, our analysis aligns closely with the performance reported in the DREAM Challenge, which aimed to identify LIDs on MJFF datasets resulting in an AUPR of 0.48. However, the latter study did not evaluate distinct data representations nor test generalization on an independent dataset [9]. Previously, the mentioned studies by Hssayeni et al. and Pfister et al. report higher performances in dyskinesia detection, but their non-clinical setups are incomparable in terms of the sensor types used or a non-standardized assessment of dyskinesia [11,12]. Moreover, almost all the presented papers reveal distinct metrics, which are incomparable to each other. As each metric analysis is favorable for the individual intention, these metrics are a challenge to evaluate in terms of comparability. To this end, we chose the F_1_ score as the principal metric for performance evaluation, as it balances precision and recall, two properties particularly important in the clinical context where both false positives and false negatives can impact therapeutic decisions.

While there is currently no universally accepted threshold for the F_1_ score in clinical ML applications, our observed values (MJFF: 0.68; PACMAN: 0.53) indicate a level of consistency and reliability that supports potential real-world use.

In order to lay the groundwork for ML generalization to work, standardization is required. First, the evaluative framework of studies working with supervised ML on movement data should use comparable and clinically relevant metrics. Analyses of medical data must account for class-specific performance, as predictions for each class need to be evaluated separately. Overall accuracy, for example, is insufficient in clinical settings, as it can obscure the detection of relevant disease phenotypes, particularly in imbalanced datasets. Secondly, the application of standardized neurological assessments, such as the suggested UPDRS, should be used. While some publications evaluate activities of daily living [11], these activities are not sufficiently reproducible and only play a minor role in therapeutic adjustments. These assessments lay the foundation of the task-specific data labeling and thus are essential for generalizability.

On the contrary, the data collection approach presented in this paper provides a unique opportunity to acquire standardized clinical assessments of movement distortions over a two-week period per patient. The dense data quality obtained per patient facilitates the detection of dyskinesias in a hospital setting enabling early identification of LIDs for medication adjustments before the patient is discharged.

Regarding clinical implementation of the presented movement data methodology, the generalizability of the fully semantic representation suggests great opportunities for future applications of wearables to detect LIDs during clinical stays. Our primary assumption, that the periodic alternation between supination and pronation of the hand are well projectable by a simple dimensional reduction, turns out to be valid. Adhering to clinical expertise and translating it directly into straightforward data representations suitable for machine learning algorithms significantly influences the final performance outcomes. Although real-time analysis was not the focus of this study, the short inference time of our trained models suggests feasibility for future real-time applications, such as adaptive therapy monitoring during inpatient stays. The dimensionally reduced representations demonstrated better generalization on the PACMAN dataset compared to the automatic features, which overfitted on the MJFF dataset. This supports the value of embedding domain knowledge into preprocessing pipelines, particularly when data availability is limited, a common challenge in real-world clinical contexts. The results on the PACMAN dataset further suggest the enormous potential for a combination of both techniques. Combining task-specific semantic dimensional reduction with automatic feature extraction may offer the best of both worlds, as this hybrid approach performed best on the PACMAN dataset. Both techniques are rooted in the assumption that the UPDRS examination, a clinically validated neurological scoring standard for over 30 years, provides a robust foundation for interpreting dyskinesia. Accordingly, the semantic pipeline partially mimics a clinician’s process of evaluating movement patterns. A multidisciplinary approach between clinical expertise and data science is imperative for a successful application of this technology into routine.

However, this technology has its limitations for the detection of dyskinesia due to its unreliable predictive power. The relatively small size of our PACMAN dataset constrains the statistical power of our findings and likely contributes to variability in model performance. This study was designed as an empirical step towards estimating minimal data requirements under clinical constraints, but future work must include larger, statistically powered datasets. Particularly, an expanded data collection of clinically annotated measurements is essential for tracking LIDs and assisting clinicians in optimizing therapy. Perhaps even synthetically generated movement data could provide a foundation to train and optimize models without the extensive need of gathering patients. Furthermore, comparability between datasets and standardization plays a pivotal role for the generalizability and its coherent ability to detect dyskinesias, as stated previously. Also, it remains unclear if other neurological assessments that are diagnostically relevant for the dyskinesia detection can be projected by semantic knowledge. This suggestion is also undermined by the smartwatch’s limitation to detect movement of fingers or the hand. Nevertheless, the outcomes of this study demonstrate potential for the development of robust decision-support systems grounded in semantic principles, particularly in the analysis of clinically recorded movement data.

## 5. Conclusions

This paper presents a unique opportunity to gather standardized clinical assessments of movement distortions over a two-week period per patient, facilitating enhanced dyskinesia detection within a hospital setting. The utilization of inbuilt accelerometers in smart watches provides a stable and convenient solution to track movement distortions in hospitalized patients. Our investigation has identified objective movement data representations conducive to dyskinesia recognition and highlighted the semantic impact on sensor data analysis, especially when generalizing onto foreign datasets. Notably, semantic feature representations demonstrated more robust performance than automated features when applied to real-world clinical data. This robustness stems from their interpretability and alignment with established clinical rating schemes. The results suggest that semantically grounded preprocessing may offer a critical advantage in small-data or high-variability scenarios. Yet, further research with a larger cohort and standardized labeling protocols is essential to optimize therapy for levodopa-induced dyskinesias. The results of this work provide evidence of feasibility, suggesting that technology-based measurements have the potential to serve as supportive tools for comprehending symptom fluctuations during clinical practice.

## Figures and Tables

**Table 1:** Results of top 5 models for representation: PCA and biomechanical features on the MJFF dataset.

Rank	Model			Macro F_1_ Score	Accuracy
	Classifier	Selector	Sampling		
1	gradient boosted trees (lr = 1.0)	10	none	0.63	0.87
2	gradient boosted trees (lr = 1.0)	none	none	0.62	0.88
3	random forest	5	SMOTE	0.61	0.89
4	gradient boosted trees (lr = 1.0)	none	SMOTE	0.60	0.83
5	k-nearest neighbors (nn = 5)	5	none	0.60	0.88

lr = learning rate, nn = number of neighbors, SMOTE = Synthetic Minority Over-sampling Technique.

**Table 2:** Results of top 5 models for representation: PCA and tsfresh features on the MJFF dataset.

Rank	Model			Macro F_1_ Score	Accuracy
	Classifier	Selector	Sampling		
1	random forest	none	SMOTE	0.68	0.88
2	random forest	10	none	0.68	0.88
3	random forest (depth = 5)	10	none	0.67	0.89
4	random forest (depth = 5)	none	SMOTE	0.67	0.84
5	gradient boosted trees (lr = 1.0)	10	none	0.66	0.86

lr = learning rate, SMOTE = Synthetic Minority Over-sampling Technique.

**Table 3:** Results of top 5 models for representation: PCA and standalone tsfresh features on the MJFF dataset.

Rank	Model			Macro F_1_ Score	Accuracy
	Classifier	Selector	Sampling		
1	random forest	10	none	0.68	0.88
2	random forest (depth = 5)	10	none	0.67	0.89
3	random forest (depth = none)	none	SMOTE	0.67	0.88
4	random forest (depth = 5)	none	SMOTE	0.67	0.83
5	random forest (depth = 5)	5	none	0.66	0.88

SMOTE = Synthetic Minority Over-sampling Technique.

**Table 4:** Results of top 5 models for representation: PCA and biomechanical features on PACMAN dataset.

Rank	Model			Macro F_1_ Score	Accuracy
	Classifier	Selector	Sampling		
1	gradient boosted trees (lr = 1.0)	none	SMOTE	0.48	0.54
2	gradient boosted trees (lr = 1.0)	10	SMOTE	0.47	0.52
3	random forest	10	SMOTE	0.45	0.53
4	gradient boosted trees (lr = 1.0)	5	none	0.44	0.53
5	gradient boosted trees (lr = 1.0)	10	none	0.41	0.51

lr = learning rate, SMOTE = Synthetic Minority Over-sampling Technique.

**Table 5:** Results of top 5 models for representation: PCA and tsfresh features on PACMAN dataset.

Rank	Model			Macro F_1_ Score	Accuracy
	Classifier	Selector	Sampling		
1	gradient boosted trees (lr = 1.0)	10	none	0.53	0.56
2	gradient boosted trees (lr = 1.0)	none	none	0.44	0.56
3	random forest	5	none	0.42	0.54
4	random forest (depth = 5)	5	none	0.40	0.54
5	random forest (depth = 5)	none	SMOTE	0.38	0.51

lr = learning rate, SMOTE = Synthetic Minority Over-sampling Technique.

**Table 6:** Results of top 5 models for representation: standalone tsfresh features on PACMAN dataset.

Rank	Model			Macro F_1_ Score	Accuracy
	Classifier	Selector	Sampling		
1	k-nearest neighbors (nn = 5)	10	none	0.37	0.54
2	random forest	10	none	0.37	0.53
3	random forest (depth = 5)	5	none	0.37	0.53
4	gradient boosted trees (lr = 1.0)	10	none	0.37	0.49
5	random forest (depth = 5)	none	SMOTE	0.36	0.52

lr = learning rate, nn = number of neighbors, SMOTE = Synthetic Minority Over-sampling Technique.

## Data Availability

The code generated during the current study is publicly available in the repository of the University of Hamburg, (http://doi.org/10.25592/uhhfdm.14189 (accessed on 16 July 2025) since 18 July 2025.

## Abbreviations

The following abbreviations are used in this manuscript:

UKE	University Medical Center Hamburg-Eppendorf
PCA	Principal Component Analysis
tsfresh	Time-Series Feature Extractors
LID	Levodopa-Induced Dyskinesia
PD	Parkinson’s Disease
MJFF	Michael J. Fox Foundation
UPDRS	Unified Parkinson’s Disease Rating Scale
LRS	Levodopa Response Study
CIS-PD	Clinician Input Study-Parkinson’s Disease
PKB	Parkinson-Komplexbehandlung
PACMAN	Parkinson’s Clinical Movement Assessment
FHIR	Fast Healthcare Interoperability Resources
ML	Machine Learning
SMOTE	Synthetic Minority Over-Sampling Technique
AUPR	Area Under the Precision-Recall Curve
DREAM	Digital Biomarker Evaluation and Analysis for Mobile Health Challenge
